# Epigallocatechin gallate protects against oxidative stress-induced mitochondria-dependent apoptosis in human lens epithelial cells

**Published:** 2008-01-31

**Authors:** Ke Yao, PanPan Ye, Li Zhang, Jian Tan, XiaJing Tang, YiDong Zhang

**Affiliations:** 1 Eye Center, Affiliated Second Hospital, College of Medicine, Zhejiang University, Hangzhou, China; 2 Department of Ophthalmology, Eye and ENT Hospital, Fudan University, Shanghai, China

## Abstract

**Purpose:**

Oxidative stress has long been recognized as an important mediator of apoptosis in lens epithelial cells and also plays an important role in the pathogenesis of cataracts. (-)-Epigallocatechin gallate (EGCG), the most abundant component in green tea, has potent antioxidant activity. The goals of this study were to determine the protective effect of EGCG against H_2_O_2_-induced apoptotic death and the possible mechanisms involved in human lens epithelial (HLE) cells.

**Methods:**

HLEB-3, a human lens epithelial cell line, was exposed to various concentrations of H_2_O_2_ and EGCG and subsequently monitored for cell death by the MTT assay and flow cytometric analysis using Annexin V and PI. The effect of EGCG in protecting HLE cells from cell death was determined by various assays after the cells were exposed to H_2_O_2_. The ability of EGCG to block the accumulation of intracellular reactive oxygen species and the loss of mitochondrial membrane potential (Δψm) induced by H_2_O_2_ was examined with dichlorofluorescein (DCF) fluorescence and 5,5′,6,6'-tetrachloro-1,1',3,3′-tetrathylbenzimidazol carbocyanine iodide (JC-1). The expression of cytochrome *c*, caspase-9, caspase-3, and Bcl-2 family proteins was measured by western blotting. The changed expression of the mitogen activated protein kinase (MAPK) and Akt pathways was also detected by western blot.

**Results:**

In the present study, EGCG protected against cell death caused by H_2_O_2_ in HLEB-3 cells. EGCG reduced the H_2_O_2_-induced generation of reactive oxygen species (ROS), the loss of mitochondrial membrane potential (Δψm), and the release of cytochrome *c* from the mitochondria into the cytosol. EGCG inhibited the H_2_O_2_-stimulated increase of caspase-9 and caspase-3 expression and the decrease of the Bcl-2/Bax ratio. Moreover, EGCG attenuated the reduced activation and expression of ERK, p38 MAPK, and Akt induced by H_2_O_2_.

**Conclusions:**

These findings suggest that EGCG protects HLE cells from the mitochondria-mediated apoptosis induced by H_2_O_2_ through the modulation of caspases, the Bcl-2 family, and the MAPK and Akt pathways.

## Introduction

Cataract formation, the opacification of the eye lens, is one of the leading causes of human blindness worldwide, accounting for 47.8% of all causes of blindness [1]. Although great advances have been made in surgical treatment, the incidence of cataracts in developing countries is so high that it overwhelms the capacity of surgical programs. Oxidative stress has long been recognized as an important mediator of apoptosis in lens epithelial cells and also plays an important role in the pathogenesis of cataracts [2-4]. The lens exists in an environment that is rich in endogenous sources of reactive oxygen species (ROS), which are produced by the high local oxygen concentration, the chronic exposure to light, and the pathogenic activities of lens epithelial cells [5]. Although multiple physiologic defenses exist to protect the lens from the toxic effects of light and oxidative damage, mounting evidence suggests that chronic exposure to oxidative stress over the long-term may damage the lens and predispose it to cataract development.

Apoptosis is a physiologic process of cell death that plays a critical role in a variety of biologic systems, which has been identified as providing an important molecular basis for both the initiation and progression of cataracts [6,7]. There are distinct mechanisms that execute apoptosis according to various different apoptotic stimuli, and these are classified into the mitochondria-dependent pathway (intrinsic pathway) and the death receptor-dependent pathway (extrinsic pathway). Previous studies have demonstrated the capacity of antioxidant protection of the mitochondria-dependent pathway associated with lens opacification in cultured lenses [8-11]. Mitochondrial damage results in the release of cytochrome *c* from the impaired mitochondria into the cytoplasm, which contributes to programmed cell death [12].

Epidemiologic data have shown that special dietary additives may provide effective defenses against oxidative stress and thus have potential as treatments for a variety of diseases. Recently, extensive studies have suggested a positive correlation between the consumption of green tea (*Camellia sinensis*) and beneficial antioxidant, anti-inflammatory, and anti-carcinogenic effects [13]. The green tea extracts contain (-)-epigallocatechin gallate (EGCG), (-)-epigallocatechin (EGC), (-)-epicatechin gallate (ECG), (-)-epicatechin (EC), and catechin [14]. EGCG, the most abundant component in green tea, has a potent antioxidant property because of two triphenolic groups in its molecular structure. Several studies have demonstrated that EGCG can protect the heart, brain, and kidney from oxidative injury [15-17]. EGCG provide both short and long-term protection against oxidative stress through a variety of mechanisms [18-20]. Although the protective effect of EGCG has been reported in various models, there are few studies about the protection of EGCG against apoptosis-related cataracts and the precise mechanism of signal transduction in this pathological condition.

Based on these observations, we hypothesize that EGCG can protect lens epithelial cells from oxidative stress-induced apoptosis and may have benefits in the treatment of cataracts associated with oxidative stress. In the work presented, we used H_2_O_2_-treated human lens epithelial (HLE) cells as a model to study lens epithelial cell exposure to oxidative stress. This study is designed to investigate the protective effect of EGCG against H_2_O_2_-induced apoptosis and the possible mechanisms involved in HLE cells.

## Methods

### Materials

HLEB-3 cells (human lens epithelial cells) were obtained from ATCC (Rockville, MD). Fetal bovine serum (FBS) and Dulbecco's modified Eagle's medium (DMEM) were obtained from Gibco (Grand Island, NY). Propidium iodide (PI), Annexin-V, EGCG, 4,5-dimethylthiazol-2-yl-2,5-diphenyltetra zolium bromide (MTT), 2',7'-dichlorofluoresceine diacetate (DCFH-DA), 5,5′,6,6'-tetrachloro-1,1',3,3′-tetrathylbenzimidazol carbocyanine iodide (JC-1), and a cocktail of protease inhibitors were from Sigma Chemical Co. (St. Louis, MO). The bicinchoninic acid (BCA) protein assay kit was from Pierce (Lockford, IL). Anti-cytochrome *c*, anti-Bax, anti-Bcl-2, anti-actin, anti-mouse, and anti-rabbit IgG horseradish peroxidase (HRP) antibodies were from Santa Cruz (Santa Cruz, CA). Anti-p-Akt (Ser 473), anti-Akt, anti-p-JNK (Thr183/Tyr185), anti-JNK, anti-p-ERK1/2, anti-ERK1/2, anti-p-p38 (Thr180/Tyr182), anti-p38, anti-pro-caspase-9, and anti-cleaved-caspase 3 antibodies were purchased from Cell Signal Technology (Beverly, MA). The chemiluminescence (ECL) detection kit was acquired from Amersham Pharmacia (Arlington Heights, IL).

### Cell culture

HLEB-3 cells were cultured in DMEM supplemented with heat-inactivated (56 °C, 0.5 h) 10% FBS at 37 °C in a humidified atmosphere of 5% CO_2_. The cells were seeded in a 60 mm culture dish (Falcon; Becton-Dickinson, Oxnard, CA). When grown to 75%–80% confluence, the cells were treated with the indicated concentration of H_2_O_2_ for the required time or pretreated with EGCG for 1 h before the H_2_O_2_ treatment. At the indicated time points, the cells were collected for the different assays.

### Cell viability assay

HLEB-3 cells were seeded in 96 well tissue culture plates at an initial concentration of 1x10^5^ cells/ml and incubated with 50, 100, and 200 μM H_2_O_2_ alone or pretreated with different concentrations of EGCG (10, 25, 50, 75, 100, and 150 μM) for 1 h. After incubation for the indicated time, cells were treated by the addition of 20 ml MTT dye to each well. After an additional 4 h incubation, the growth medium was removed and the formazan crystals, formed by oxidation of the MTT dye, were dissolved with 150 μl DMSO in isopropanol. The absorbance was measured at 490 nm and the cell survival ratio was expressed as a percentage of the control.

### Flow cytometric analysis using annexin V and PI

Cells were grown on a six-well plate at 1x10^6^ cells per plate and treated with 100 μM H_2_O_2_ for 24 h. For 50 μM EGCG, cells were pretreated for 1 h before treatment. Cells were centrifuged to remove the medium, washed with PBS, and stained with annexin V-FITC and PI in binding buffer (10 mM Hepes, 140 mM NaCl, and 2.5 mM CaCl_2_). Ten thousand events were collected for each sample. Stained cells were analyzed using a FACS calibur (Becton Dickinson, Mountain View, CA) in the FL1-H and FL2-H channels.

### Detection of reactive oxygen species

The production of reactive oxygen species (ROS) was monitored using flow cytometry. Cells were plated on a six-well plate and pretreated with 50 μM EGCG for 1 h followed by treatment with 100 μM H_2_O_2_ for 2 h. DCFH-DA (10 μM) was added into the medium for 15 min at 37 °C. The intracellular production of ROS was determined with excitation at 480 nm and with emission at 530 nm.

### Measurement of mitochondrial membrane potential (Δψm)

The cells were incubated with 100 μM H_2_O_2_ for 6 h or pretreated with 50 μM EGCG for 1 h in the presence of H_2_O_2_. The Δψm of cells was measured by staining with JC-1. When mitochondria were polarized, JC-1 formed aggregates and emitted red fluorescence with 486 nm excitation. The red and green fluorescence were measured simultaneously by FACS caliber.

### Immunoblot analysis

After treatment, cell cultures were washed once in cold PBS and then lysed in a lysis buffer containing 50 mM Tris-HCl, pH 7.5, 150 mM NaCl, 1 mM Na_2_EDTA, 1 mM EDTA, 1% Triton, 2.5 mM sodium pyrophosphate, 1 mM β-glycerophosphate, 1 mM Na_3_VO_4_, 1 mM NaF, 1 μg/mL leupeptin, and 1 mM PMSF. The protein extracts were quantified using the bicinchoninic acid (BCA) protein assay kit (Pierce). The samples containing 20–40 μg proteins were boiled in Laemmli sample buffer, electrophoresed in 10% sodium dodechyl sulfate-polyacrylamide gels (SDS–PAGE), and transferred onto polyvinylidene difluoride (PVDF) membranes (Millipore, Milford, MA) in trans-blot transfer medium (20 mM Tris-HCl, 190 mM Glycine, 20% methanol, pH 8.3). All membranes were blocked in TBS buffer (10 mM Tris-HCl, 50 mM NaCl, and 0.05% Tween 20) containing 5% BSA for 1 h at room temperature, and all subsequent antibody dilutions and washings were performed with TBS buffer. Membranes were incubated with the following antibodies overnight at 4 °C: anti-cytochrome *c*, anti-Bax, anti-Bcl-2, anti-pro-caspase-9, anti-cleaved-caspase-3, anti-p-Akt, anti-Akt, anti-p-p38 MAPK, anti-p38 MAPK, anti-p-ERK, anti-ERK, anti-p-JNK, anti-JNK, and anti-actin. Membranes were washed three times in TBS buffer, incubated with HRP-conjugated secondary antibodies for 1 h at room temperature, then washed and visualized by chemiluminescence.

### Statistical analysis

Data are expressed as the mean±SD. Statistical analysis of the data was performed using SigmaStat software (Jandel Scientific Software, San Rafael, CA). Comparison between groups was made using one-way ANOVA followed by the Student–Newman–Keuls test. A p-value of less than 0.05 was considered significant.

## Results

### (-)-epigallocatechin gallate protects against H_2_O_2_-induced cell death in HLEB-3 cells

**Figure 1:**
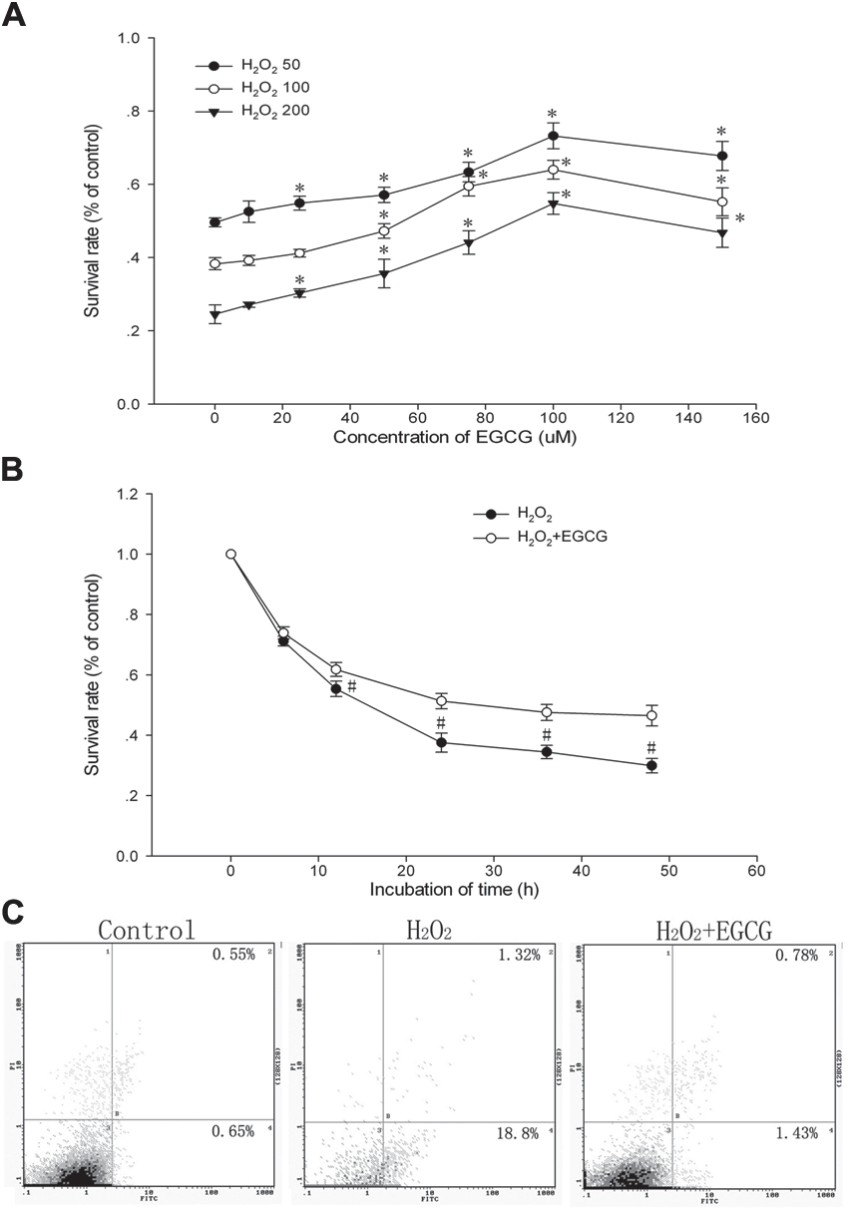
(-)-epigallocatechin gallate protects against H_2_O_2_-induced cell death in HLEB-3 cells. **A**: After the HLEB-3 cells were incubated with different concentrations of H_2_O_2_ (50, 100, and 200 μM) for 24 h or with EGCG treatment (10, 25, 50, 75, 100, and 150 μM) 1 h before H_2_O_2_ treatment, the viability was measured by MTT assay. **B**: The cell viability pretreated with EGCG at 50 μM for 1 h before exposure to 100 μM H_2_O_2_ was estimated using MTT assay with a time-course. The data are represented as mean±SD from three independent experiments. The asterisk indicates that p<0.05 compared to the untreated control and the sharp (hash mark) means that p<0.05 compared to the H_2_O_2_-treated group. **C**: The cells were incubated with 50 μM EGCG for 1 h then treated with 100 μM H_2_O_2_ for 24 h. The result is one representative example of three separate experiments.

The oxidant H_2_O_2_ has been used in the design of models of classical oxidative stress in HLEB-3 cells because of its rapid membrane permeability and depolarizing effects on the mitochondrial membrane potential. For these reasons, we selected it for our studies and performed a series of dose- and time-response assays to determine the working concentration and time that led to a consistent degree of cytotoxicity. The cells were exposed to different concentrations of EGCG (10, 25, 50, 75, 100, and 150 μM) for 24 h, and the MTT assay showed no loss of cell viability (data not shown). Treatment with H_2_O_2_ at 50, 100, and 200 μM for 24 h caused a dose-dependent loss of viability (50.0%±1.2%, 38.3%±1.7%, and 24.5%±2.6% of control value) whereas the EGCG pretreatment at a concentration from 10 to 150 μM for 1 h prevented the loss of viability (Figure 1A). The viability of cells pretreated with EGCG at 100 μM for 1 h before exposure to 100 μM H_2_O_2_ was up to 64.0%±2.6% whereas the increased concentration of 150 μM did not cause any enhancement of this preventive effect. We analyzed the effective half maximal concentration for protection (EC_50_s) from the dose–response curves and decided to adopt the concentration of 50 μM for our subsequent studies. Moreover, the application of 100 μM H_2_O_2_ induced time-dependent cytotoxicity which was prevented by EGCG in a time-dependent manner (Figure 1B).

To examine whether EGCG protects against H_2_O_2_-induced apoptosis, the HLEB-3 cells were incubated with 50 μM EGCG for 1 h then treated with 100 μM H_2_O_2_ for 24 h. Flow cytometric analysis was used to quantify the rate of cell apoptosis using double staining of Annexin V-FITC and PI. A significant increase of apoptosis was observed in HLEB-3 cells treated with 100 μM H_2_O_2_ compared with control cells (20.12% versus 1.2%; Figure 1C). However, EGCG-pretreated HLE cells showed significant resistance to H_2_O_2_-induced apoptosis (2.2%).

### (-)-epigallocatechin gallate reduces the generation of reactive oxygen species and prevents the loss of Δψm and the release of mitochondrial cytochrome *c* into the cytosol in HLEB-3 cells

**Figure 2:**
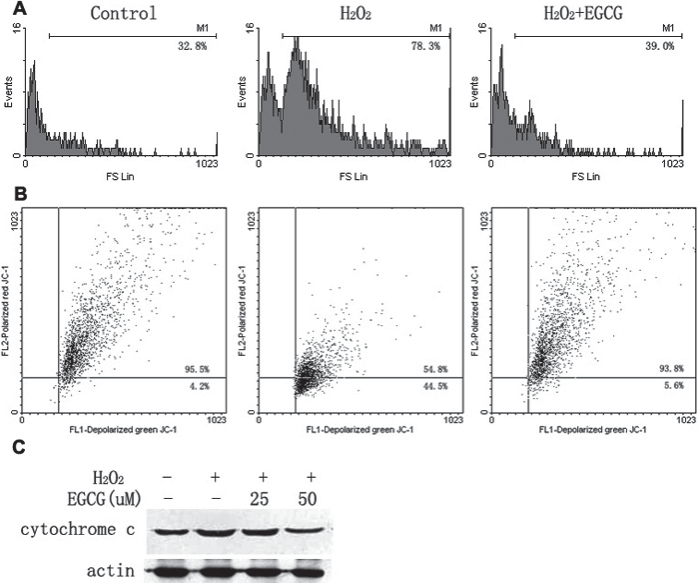
(-)-epigallocatechin gallate reduces the generation of ROS and prevents the loss of Δψm and the release of mitochondrial cytochrome *c* into the cytosol in HLEB-3 cells. **A**: The cells were pretreated with 50 μM EGCG for 1 h followed by treatment with 100 μM H_2_O_2_ for 2 h. The production of ROS was examined by measuring the level of ROS production using DCF-DA by flow cytometry. **B**: The cells were incubated with either 100 μM H_2_O_2_ for 6 h or pretreated 50 μM EGCG for 1 h in the presence of H_2_O_2_. The Δψm of cells was measured by staining with JC-1 by flow cytometry. **C**: After treatment for 24 h with 100 μM H_2_O_2_ or with 25 and 50 μM EGCG in the presence of H_2_O_2_, the cells were analyzed by western blot analysis. The result is one representative example of three separate experiments.

The ROS induced by H_2_O_2_ were examined by measuring the level of ROS production using DCF-DA. HLEB-3 cells treated with 100 μM H_2_O_2_ for 2 h resulted in the production of ROS with an approximately twofold increase compared to nontreated cells (Figure 2A). However, pretreatment with EGCG at 50 μM before H_2_O_2_ exposure markedly reduced the ROS levels generated by H_2_O_2_ in HLEB-3 cells.

Loss of Δψm is an early event of apoptosis induced by a variety of stimuli. To examine whether the Δψm pathway is involved in H_2_O_2_-induced apoptosis or is changed by EGCG in the presence of H_2_O_2_, Δψm detection was performed using dye JC-1, which was used to assess mitochondrial membrane depolarization. As shown in Figure 2B, cells treated with H_2_O_2_ exhibited a substantial increase in mitochondrial depolarization; the percentage of cells testing positive for depolarized mitochondria increased from 4.2% of control cells to 44.5% of cells treated with 100 μM H_2_O_2_. Depolarization of mitochondria releases several apoptogenic proteins, most notably cytochrome *c*, from the mitochondria into the cytosol. Western blot revealed that 100 μM H_2_O_2_ led to an accumulation of cytochrome *c* in the cytosol for 24 h (Figure 2C). The observed cytochrome *c* in the cystol was significantly reduced when the cells were pretreated with 50 μM EGCG before H_2_O_2_ treatment. These results indicate that H_2_O_2_ causes a mitochondrial dysfunction via the mitochondrial pathway and EGCG is protective against such dysfunction.

### (-)-epigallocatechin gallate inhibits the activities of caspase-9 and caspase-3 and modulates the expression of the Bcl-2 family proteins induced by H_2_O_2_ in HLEB-3 cells

**Figure 3:**
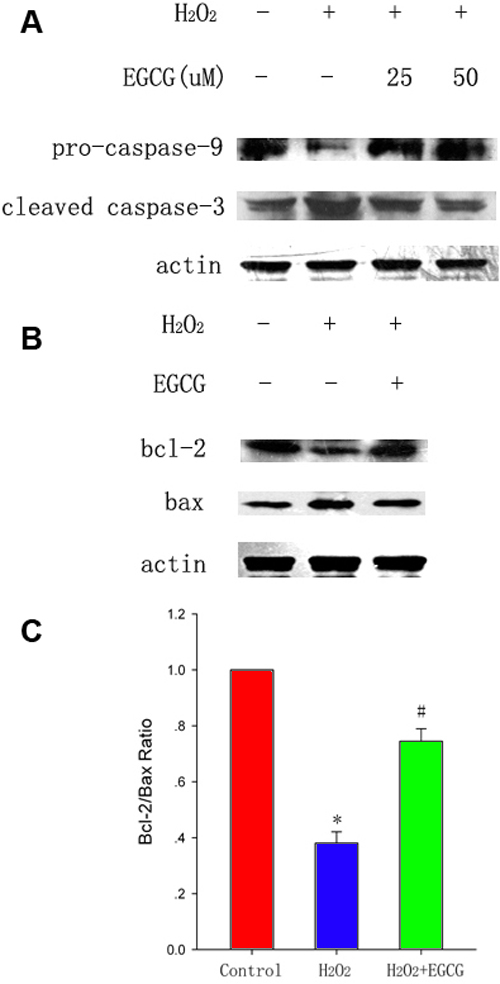
(-)-epigallocatechin gallate inhibits the activities of caspase-9 and caspase-3 and modulates the expression of the Bcl-2 family proteins induced by H_2_O_2_ in HLEB-3 cells. The cells were treated with 25 and 50 μM EGCG in presence of 100 μM H_2_O_2_ for 24 h. **A**: The expression of caspase-9 and caspase-3 mRNA was detected by RT–PCR. **B**: The expression of the pro-caspase-9 and cleaved-caspase-3 protein was analyzed by western blot analysis. **C**: Western blot was performed for Bcl-2 and Bax expression. **D**: Densitometric analyses of western blot are presented as the mean±SD for three independent experiments performed in triplicate. Data are presented as the fold induction over control cells. The asterisk indicates that p<0.05 compared to the untreated control and the sharp (hash mark) means that p<0.05 compared to the H_2_O_2_-treated group.

Caspases are known to play important roles in apoptosis. To examine whether caspases are activated in H_2_O_2_-induced apoptosis, we examined the expression of caspase-9 and caspase-3 at the protein level. Western blot analysis revealed a decrease of pro-caspase-9 as a result of caspase-9 activation in H_2_O_2_-treated cells, which was prevented by a pretreatment with EGCG (Figure 3A). The decrease in pro-caspase-9 was associated with increased caspase-3 cleavage with 17 kDa and 19 kDa, a downstream substrate of caspase-9. The effect of H_2_O_2_-induced caspase-3 cleavage was also rescued by EGCG.

Either the pro-apoptotic or anti-apoptotic Bcl-2 family members can affect the execution of apoptosis. To determine whether EGCG protects against H_2_O_2_-induced apoptosis in HLEB-3 cells by modulating Bcl-2 family, the protein levels of Bax and Bcl-2 were examined by western blot analysis (Figure 3B). Treatment with 100 μM H_2_O_2_ decreased the expression of Bcl-2 and increased the expression of Bax while pretreatment with EGCG inhibited downregulation of Bcl-2 and upregulation of Bax. The ratio of Bcl-2 to Bax was significantly reduced by H_2_O_2_ and attenuated by pretreatment with EGCG (Figure 3C).

### (-)-epigallocatechin gallate protects against H_2_O_2_-induced apoptosis through activation of mitogen activated protein kinases and Akt

**Figure 4:**
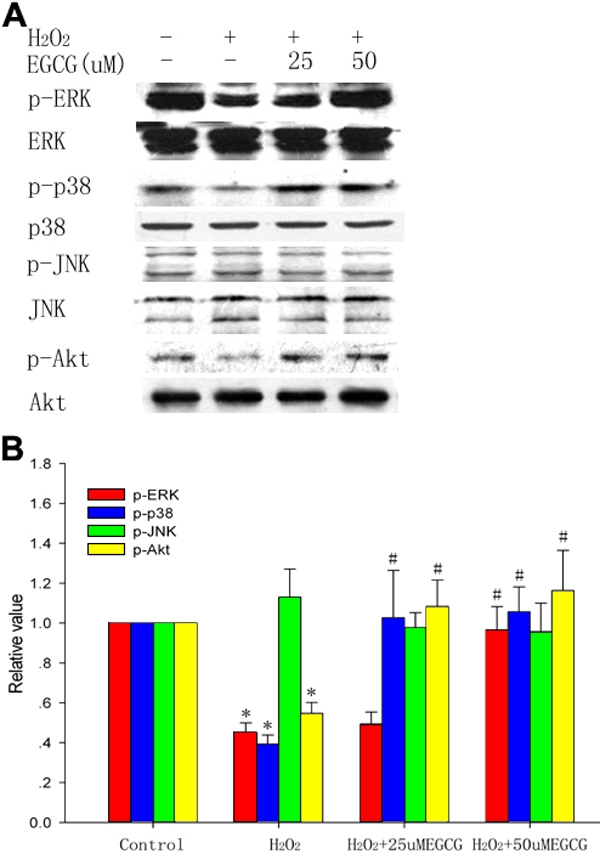
(-)-epigallocatechin gallate protects against H_2_O_2_-induced apoptosis through activation of the MAPKs and Akt pathways. **A**: After the cells were incubated for 24 h with 100 μM H_2_O_2_ or with 25 and 50 μM EGCG 1 h before H_2_O_2_ treatment, the expression of ERK, p38 MAPK, JNK, and Akt protein was detected using western blot analysis. **B**: Densitometric analyses of western blot are presented as the mean±SD for three independent experiments performed in triplicate. Data are presented as the fold induction over control cells. The asterisk indicates that p<0.05 compared to the untreated control and the sharp (hash mark) means that p<0.05 compared to the H_2_O_2_-treated group.

To gain further insight into the molecular mechanisms involved, we studied the effect of EGCG on the possible pathways potentially activated during apoptosis. Therefore, we investigated whether the MAPKs and Akt pathways were induced by treatment with or without EGCG. As shown in Figure 4, stimulation with H_2_O_2_ resulted in a significant decrease in the phosphorylated forms of ERK (p44 and p42) and p38 MAPK and Akt (Ser 473) whereas there was no change in total ERK, p38, or Akt expression. The H_2_O_2_-induced downregulation of ERK and p38 MAPK and Akt was significantly inhibited by pretreatment with EGCG at concentrations of 25 and 50 μM, respectively. However, JNK was not activated by treatment with H_2_O_2_ although this kinase was present in the cells.

## Discussion

Cellular defenses that protect the lens epithelial cells against oxidative stress have been proposed to be an important way to reduce the progression of various types of cataracts [21]. Previous studies have demonstrated that H_2_O_2_-induced apoptosis in HLE cells is a useful model for studying cataractogenesis [22,23]. Although EGCG has been reported to protect against oxidative stress in other human cell lines [24,25], little had been demonstrated before this study in terms of its effect in lens epithelial cells. In this study, EGCG remarkably inhibited the cytotoxicity of HLE cells caused by H_2_O_2_ in a range of 10–100 μM compared to treatment with H_2_O_2_ alone. However, EGCG at a concentration 150 μM did not cause any increase of cell viability. One possible reason is the cell toxicity due to the accumulation of the two drugs at high concentrations. Moreover, flow cytometry analysis using PI and a nnexin V showed that H_2_O_2_ induced cell apoptosis in HLE cells whereas EGCG significantly reduced the apoptosis in H_2_O_2_-treated cells. This indicates that EGCG at low concentrations exerts a protective effect by inhibiting H_2_O_2_-induced cell death.

Oxidative stress can disrupt the balance between reactive oxygen radical production and the radical scavenging effect and lead to apoptotic cell death through the mitochondrial apoptosis pathway. Previous studies have shown mitochondrial protection to be important in the process of lens opacification in cultured lenses [8,10,11]. The collapse of the mitochondrial membrane potential results in the rapid release of cytochrome *c* into the cytoplasm [12]. Although the mechanism is still not completely understood, EGCG has recently been recognized to scavenge intracellular ROS and regulate antioxidant enzyme activities [26]. Consistent with these findings, H_2_O_2_-treated cells showed an increased production of intracellular ROS, a loss of mitochondrial potential, and an increased release of mitochondrial cytochrome *c* into the cytoplasm. However, pretreatment with EGCG attenuated the increase in ROS level and prevented the loss of mitochondrial potential and the release of cytochrome *c*. These results indicate H_2_O_2_ is an oxidative stress which stimulates the production of ROS and triggers the mitochondria-mediated apoptosis pathway, and these effects can be suppressed by EGCG.

Caspases play an important role in regulating cell apoptosis. Caspases transduce the apoptotic signal cascade and engage cellular targets leading to programmed cell death [27,28]. As one of the key effectors, caspase-3 is initiated by caspase-9 and involved in the mitochondria-mediated pathway. Previous studies have shown that H_2_O_2_ induced the activation of caspase-9 and caspase-3 in lens epithelial cells [23,29]. Consistent with these results, we demonstrated that the induction of apoptosis induced by H_2_O_2_ was accompanied by an increase of caspase-9 and caspase-3 activities at the protein level. Furthermore, this effect could be attenuated by a pretreatment with EGCG before H_2_O_2_ treatment. Caspase-9 and caspase-3 might be important effector caspases in H_2_O_2_-induced apoptosis, and EGCG protects against the apoptosis of HLE cells by blocking the expression of caspase-9 and caspase-3.

The Bcl-2 family, which possesses both anti- and pro-apoptotic members, constitutes a decisive checkpoint within the common portion of the cell death pathway [30]. Bcl-2 can prevent ROS production and regulate the mitochondrial transitional pore opening by opposing the effect of Bax thereby blocking cytochrome *c* release and inhibiting caspase activities [31]. An altered ratio of anti-apoptotic/pro-apoptotic Bcl-2 family proteins is critical in determining whether apoptosis is performed. In this study, western blot analysis revealed that the Bcl-2/Bax ratio was significantly decreased by the treatment with H_2_O_2_, and this decrease was inhibited by the pretreatment with EGCG. This result indicates that Bcl-2 family proteins may play a critical role in regulating HLE cell death induced by H_2_O_2_, and EGCG is able to protect against H_2_O_2_-stimulated apoptosis through a modulation of Bcl-2/Bax expression.

Several investigations have reported that EGCG can modulate growth and survival in many tumor cells through an activation of intracellular signaling cascades such as the mitogen activated kinase pathway (MAPK) and phosphoinositol-3-kinase/Akt (PI3K/Akt) pathway [32,33]. Both of these pathways have roles in anti-apoptotic and growth stimulatory signaling. To detect the pathways, which are involved in the protection afforded by EGCG against oxidative stress, we examined the expression of three major signaling proteins in the MAPKs and Akt pathways. Our data showed that H_2_O_2_ significantly decreased the activation of ERK, p38 MAPK, and Akt but not the expression of JNK whereas pretreatment with EGCG inhibited the downregulation of these proteins. This implies that EGCG may offer protection against oxidative stress-induced cell death by regulating the MAPKs and Akt pathways.

Taken together, the present study suggests H_2_O_2_ to be an oxidative stress which can induce HLE cell apoptosis through mitochondrial pathway. EGCG, a potent antioxidant, protects against H_2_O_2_-induced apoptosis by regulating caspases, the Bcl-2 family, and the MAPKs and Akt pathways. It may be exploited as a potentially useful method for cataract prevention.
